# Reported Barriers to Healthcare Access and Service Disruptions Caused by COVID-19 in Burkina Faso, Ethiopia, and Nigeria: A Telephone Survey

**DOI:** 10.4269/ajtmh.20-1619

**Published:** 2021-06-23

**Authors:** Nega Assefa, Ali Sié, Dongqing Wang, Michelle L. Korte, Elena C. Hemler, Yasir Y. Abdullahi, Bruno Lankoande, Ourohiré Millogo, Angela Chukwu, Firehiwot Workneh, Phyllis Kanki, Till Baernighausen, Yemane Berhane, Wafaie W. Fawzi, Ayoade Oduola

**Affiliations:** 1 College of Health and Medical Sciences, Haramaya University, Harar, Ethiopia;; 2 Nouna Health Research Center, Nouna, Burkina Faso;; 3 Department of Global Health and Population, Harvard T.H. Chan School of Public Health, Harvard University, Boston, Massachusetts;; 4 Jegula Hospital, Harar, Ethiopia;; 5 Institut Supérieur des Sciences de la Population, University of Ouagadougou, Ouagadougou, Burkina Faso;; 6 Department of Statistics, University of Ibadan, Ibadan, Nigeria;; 7 Department of Epidemiology and Biostatics, Addis Continental Institute of Public Health, Addis Ababa, Ethiopia;; 8 Department of Immunology and Infectious Diseases, Harvard T.H. Chan School of Public Health, Harvard University, Boston, Massachusetts;; 9 Heidelberg Institute of Global Health, University of Heidelberg, Heidelberg, Germany;; 10 Africa Health Research Institute, KwaZulu-Natal, South Africa;; 11 Department of Nutrition, Harvard T.H. Chan School of Public Health, Harvard University, Boston, Massachusetts;; 12 Department of Epidemiology, Harvard T.H. Chan School of Public Health, Harvard University, Boston, Massachusetts;; 13 University of Ibadan Research Foundation, University of Ibadan, Ibadan, Nigeria

## Abstract

The coronavirus disease 2019 (COVID-19) pandemic may have short-term and long-term impacts on health services across sub-Saharan African countries. A telephone survey in Burkina Faso, Ethiopia, and Nigeria was conducted to assess the effects of the pandemic on healthcare services from the perspectives of healthcare providers (HCPs) and community members. A total of 900 HCPs (300 from each country) and 1,797 adult community members (approximately 600 from each country) participated in the study. Adjusted risk ratios (ARRs) and 95% confidence intervals (CIs) were computed using modified Poisson regression. According to the HCPs, more than half (56%) of essential health services were affected. Child health services and HIV/surgical/other services had a slightly higher percentage of interruption (33%) compared with maternal health services (31%). A total of 21.8%, 19.3%, and 7.7% of the community members reported that their family members and themselves had difficulty accessing childcare services, maternal health, and other health services, respectively. Nurses had a lower risk of reporting high service interruptions than physicians (ARR, 0.85; 95% CI, 0.56–0.95). HCPs at private facilities (ARR, 0.71; 95% CI, 0.59–0.84) had a lower risk of reporting high service interruptions than those at governmental facilities. Health services in Nigeria were more likely to be interrupted than those in Burkina Faso (ARR, 1.38; 95% CI, 1.19–1.59). Health authorities should work with multiple stakeholders to ensure routine health services and identify novel and adaptive approaches to recover referral services, medical care, maternal and child health, family planning, immunization and health promotion, and prevention during the COVID-19 era.

## INTRODUCTION

The world has dramatically changed since a new viral illness called the coronavirus disease 2019 (COVID-19) emerged.^1^ The global burden of COVID-19 is changing; recently, more than 73 million individuals have contracted the virus, and more than 500,000 have died.^2^ In Africa, more than two million established COVID-19 cases and 50,000 deaths were reported on August 15, 2020, with 48,665 total cases in Nigeria, 27,242 total cases in Ethiopia, and 1237 total cases in Burkina Faso.^3,4^ To stop its spread, national governments and international organizations have introduced lockdown measures, strategies, and guidelines for infection control, including social distancing and self-isolation, severely restricting and affecting daily life.^5^ Such strategies worsened the accessibility of routine health services because of the sudden freezing of economic activities and significant adverse impact on income across various employment categories.^6^ This left vulnerable groups at risk for preventable diseases and complications, especially in sub-Saharan Africa.^7^

COVID-19 has significant effects on all functions of the healthcare system. Countries had to reorganize resources to deliver health services, prevent transmission, and protect healthcare providers (HCPs) and patients.^8^ Hospitals in many areas have been under intense pressure while caring for an increasing number of infected individuals requiring intensive care and simultaneously facing shortages of mechanical ventilators and personal protective equipment.^9^ In sub-Saharan Africa, some countries like Ethiopia and Nigeria had clear health service provision inequality, inequity, and disparities in accessing essential primary healthcare and skilled HCPs before COVID-19.^10,11^ Considering the weak health systems and limited resources, the COVID-19 pandemic further burdened the healthcare system and worsened the availability and disparity of essential health services.^12–14^ Subsequently, shortages of HCPs, a lack of guidelines regarding the continuation of non-COVID-19 services, and discouragement among HCPs because of the lack of equipment and materials have created challenging situations in many healthcare settings.^15^ There are also consequences for sustaining health programs that are primarily donor-funded, such as those that prevent and treat malaria, HIV/AIDS, and tuberculosis.^7,16^

The United Nations Children’s Fund (UNICEF) projected a 30% reduction in overall essential nutrition services coverage at the start of the pandemic.^17^ This reduction in access to healthcare and general disruption are expected to have devastating effects, including a significant increase in child and maternal deaths.^18^ An increase in the death rate by 10% is also anticipated in countries with higher HIV/AIDS burdens because of the COVID-19-related interruption of the medical supply chain.^14^ Many studies have also reported a decrease in maternal, sexual and reproductive, surgical, and community pharmaceutical provisions of health services. Moreover, countries face increased domestic abuse and sex inequality.^19–22^ Furthermore, increased inappropriate prescriptions of antibiotics to treat COVID-19 are expected to result in the emergence of drug-resistant bacteria.^23^

The effects of COVID-19 on health services are not well-documented, and such evidence is critical to enable the planning of services to avoid preventable mortality and morbidity. This study aimed to characterize the impacts of the COVID-19 pandemic on the interruptions on health services from the perspectives of both HCPs and community members in three sub-Saharan African countries, Burkina Faso, Ethiopia, and Nigeria.

## MATERIALS AND METHODS

### Study settings.

This study was performed in three sub-Saharan African countries, Burkina Faso, Ethiopia, and Nigeria. It aimed to collect data regarding the effects of COVID-19 on the healthcare system from the perspectives of two groups of stakeholders: healthcare providers and community members. The survey for healthcare providers was performed in Ouagadougou (Burkina Faso), Addis Ababa (Ethiopia), and Lagos and Ibadan (Nigeria). The survey for community members was conducted among households in urban and rural areas of each country (Nouna and Ouagadougou in Burkina Faso, Kersa and Addis Ababa in Ethiopia, Lagos, and Ibadan in Nigeria). Detailed methods and descriptions of the study site are reported elsewhere.^24^

### Study design.

This telephone survey was performed from July to November 2020, among healthcare workers working in major urban areas. The telephone numbers for the healthcare providers were accessed through public repositories and professional associations. The household survey telephone numbers were accessed through existing Health and Demographic Surveillance Sites (HDSS), previous surveys, and household censuses. The data collectors interviewed HCPs and household adults from virtual call centers. During this survey, among the healthcare providers, only medical physicians and nursing staff were included. The procedures of contacting institutions and individuals, identifying respondents, and implementing the study protocol are described in detail elsewhere.^24^ Distinctions were made between governmental and private facilities that provide secondary/tertiary care and the facilities that provide primary care (health outposts and health centers).

The study included 300 HCPs in each country and 900 in total. The community survey in each country included approximately 300 adult residents at the urban site and approximately 300 adult residents at the rural site (600 from each country). The study rationale, sampling strategies, and the use of computer-assisted telephone interviewing (CATI) technology during the study are described in detail elsewhere.^24^

All research staff members were trained regarding study procedures, including screening, consent, enrollment, and data collection; the importance of safeguarding the study participants’ rights and well-being was emphasized. Experts translated the consent script and study instruments to the local languages of the respective countries. The data collectors obtained informed consent electronically and verbally, and they used standardized electronic questions when communicating with the participants. Data collectors were trained to ensure the confidentiality of healthcare providers and community participants. This survey was approved by ethical review boards at Harvard T.H. Chan School of Public Health and in each country, including the Nouna Health Research Center Ethical Committee and National Ethics Committee in Burkina Faso, the Institutional Ethical Review Board of Addis Continental Institute of Public Health in Ethiopia, the University of Ibadan Research Ethics Committee, and the National Health Research Ethics Committee in Nigeria.

### Data analysis.

The healthcare providers’ responses were analyzed individually and descriptive statistics were summarized for each country. The provisions of several essential health services from the perspective of the HCPs were examined. Questions related to the interruption of a type of health service were scored from 0 to 2 (0 = no interruption; 1 = partial interruption; 2 = complete interruption). The four components of child health services were immunization, vitamin A supplementation, preventive nutrition service, and malnutrition management. The three components of maternal and reproductive health services were antenatal care, iron and folate supplementation, and family planning services. The three components of other healthcare services were HIV treatments, surgical services, and all other health services. The scores for child health services, maternal and reproductive health services, and other health services were 8, 6, and 6, respectively.^17,18^ A total score (range, 0–20) for essential service interruption was also created by summing the scores for the 10 individual services. We defined the total interruption score as high when it was more than the average score.^17,18^

Access to essential health services was examined for the community participants using 10 questions. They were asked about the difficulty accessing a specific type of healthcare by themselves or by their immediate family members. Each question elicited a yes or no response and scored as 0 (for no) or 1 (for yes). The mean score was used as a cutoff point to define reduced access. Responses were unscored if the services were not applicable or if participants refused to answer the question. The maximum score for child health services was 4 (1 point each for immunization, malnutrition treatment, vitamin A, and nutrition preventive services). For maternal and reproductive health and other health services, the maximum score was 3 (1 point each for antenatal care, iron and folic acid supplementation, and sexual and reproductive health services). For other essential health services, the maximum score was 3 (1 point each for HIV treatment, surgeries, and others). For comprehensive essential services, the maximum score was 10, including the three components already noted.^25,26^

We calculated the means and standard deviations (SDs) for normally distributed continuous variables and counts and proportions for categorical variables. Predictors of high service interruptions were examined by the modified Poisson regression^27,28^ using demographic characteristics as potential predictors. Crude risk ratios (CRRs) and adjusted risk ratios (ARRs) were calculated with 95% confidence intervals (CIs). Statistical analyses were conducted using Stata version 16 (Stata Corp., College Station, TX) with a two-sided significance level of 0.05.

## RESULTS

### Sociodemographic characteristics of the participants.

A total of 900 HCPs participated in this telephone survey; their average age was 39.8 years. Physicians accounted for 31% of the HCP participants, and females constituted 59% of the HCP participants. All HCPs from Ethiopia and Nigeria worked at governmental or private facilities that provided secondary or tertiary healthcare; however, in Burkina Faso, 23% worked at health outposts and centers that provided primary healthcare. Most of the Ethiopian participants (64%) had treated COVID-19 patients, but only 14% in Burkina Faso and 43% in Nigeria had treated them. Most participants in Burkina Faso (83%) and Nigeria (98%) reported having COVID-19 guidelines at their facility, whereas only 61% of the HCPs in Ethiopia had such guidelines (Table 1).

**Table 1:** Sociodemographic characteristics of healthcare providers in three sub-Saharan African countries

	Burkina Faso	Ethiopia	Nigeria	
	Ouagadougou	Addis Ababa	Lagos	Total
Age, years†	39.73/37.50 (9.91/25–75)	34.40/30.00 (10.53/21–72)	45.18/45.00 (9.09/23–77)	39.77/39.00 (10.79/21–77)
Sex*				
Male	157 (52.33)	141 (47.00)	74 (24.67)	372 (41.33)
Female	143 (47.67)	159 (53.00)	226 (75.33)	528 (58.67)
Occupation*				
Physician	81 (27.00)	120 (40.00)	77 (25.67)	278 (30.89)
Nurse and other‡	219 (73.00)	180 (60.00)	223 (74.00)	622 (69.11)
Facility*				
Government hospital/clinic	161 (53.67)	211 (70.33)	255 (85.00)	627 (69.67)
Private hospital/clinic	71 (23.67)	89 (29.67)	45 (15.00)	205 (22.78)
Other§	68 (22.67)	0 (0.00)	0 (0.00)	68 (7.56)
Treated COVID-19 patients*				
Yes	41 (13.67)	192 (64.00)	130 (43.33)	363 (40.33)
No	259 (86.33)	108 (36.00)	170 (56.67)	537 (59.67)
Workplace COVID-19 guidelines*	249 (83.00)	182 (60.67)	295 (98.33)	726 (80.67)

* Number of observations (%).

† Mean (standard deviation)/median (range).

‡ Clinical officers and community health workers.

§ Health outposts and health centers that provide primary healthcare (as opposed to government or private hospitals/clinics that provide secondary or tertiary healthcare).

### COVID-19 and health service interruption from the perspective of HCPs.

Many HCPs reported partial or complete interruptions to immunization and malnutrition management services during the COVID-19 pandemic. More than 50% of HCPs reported partial or complete interruptions of vitamin A supplementation, malnutrition management, and nutrition health services. Similarly, across all sites, more than 50% had interrupted antenatal care, folate supplementation, and family planning. The percentages reporting interruptions of child health services in Burkina Faso, Ethiopia, and Nigeria were 32%, 28%, and 39%, respectively. For maternal health services, the mean interruption score in Nigeria (2.24 out of 6) was higher than that in Burkina Faso (1.72 out of 6) and Ethiopia (1.67 out of 6). In general, almost all health services were reported to have experienced at least partial interruption. Except for other services (outpatient clinics, referral, tuberculosis, and cancer care) and immunization services, the proportion of interruption was not statistically different across the countries. The mean interruption score of the child healthcare service components was higher statistically than those of the maternal and other healthcare services, and all healthcare services had statistically different mean scores across the sites (Table 2).

**Table 2:** Healthcare providers’ responses to questions regarding the effects of COVID-19 on healthcare services in three sub-Saharan African countries

Services		Range of score	Burkina Faso	Ethiopia	Nigeria	Total
			Ouagadougou	Addis Ababa	Lagos	
Child health services	Immunization*¶	0–2
	No interruption		132 (44.00)	160 (53.33)	69 (23.00)	361 (40.10)
	Partial interruption		144 (48.00)	112 (37.33)	219 (73.00)	475 (52.78)
	Complete interruption		24 (8.00)	28 (9.30)	12 (4.00)	64 (7.11)
	Vitamin A supplementation	0–2
	No interruption		136 (45.33)	159 (53.00)	74 (24.67)	369 (41.00)
	Partial interruption		139 (46.33)	114 (38.00)	217 (72.30)	470 (52.22)
	Complete interruption		25 (8.33)	27 (9.00)	9 (3.00)	61 (6.78)
	Nutrition preventive services*	0–2
	No interruption		135 (45.00)	156 (52.00)	72 (24.00)	363 (40.33)
	Partial interruption		144 (48.00)	120 (40.00)	219 (73.00)	483 (53.67)
	Complete interruption		21 (7.00)	24 (8.00)	9 (3.00)	54 (6.00)
	Malnutrition management*	0–2
	No interruption		137 (45.67)	154 (51.3)	77 (25.67)	368 (40.89)
	Partial interruption		140 (46.67)	122 (40.67)	215 (71.67)	477 (53.00)
	Complete interruption		23 (7.00)	24 (8.00)	8 (2.67)	55 (6.10)
	Total interruption score‡¶ (% with interruption)†	0–8	2.51 (31.75)	2.25 (28.12)	3.15 (39.37)	2.63 (32.87)
Maternal and reproductive healthcare	Antenatal care*	0–2
	No interruption		148 (49.17)	161 (53.67)	82 (27.33)	391 (43.44)
	Partial interruption		131 (43.67)	115 (38.33)	212 (70.67)	458 (50.89)
	Complete interruption		21 (7.00)	24 (8.00)	6 (2.00)	51 (5.67)
	Iron and folic supplementation*	0–2
	No interruption		145 (48.17)	163 (54.33)	85 (28.33)	393 (43.67)
	Partial interruption		134 (44.67)	117 (39.00)	209 (69.67)	460 (51.11)
	Complete interruption		21 (7.00)	20 (6.67)	6 (2.00)	47 (5.22)
	Family planning*	0–2
	No interruption		147 (49.00)	158 (52.67)	80 (26.67)	385 (42.87)
	Partial interruption		138 (46.00)	121 (40.33)	214 (71.33)	473 (52.56)
	Complete interruption		15 (5.00)	21 (7.00)	6 (2.00)	42 (4.67)
	Total interruption score‡¶ (% with interruption)†	0–6	1.72 (28.67)	1.67 (27.83)	2.24 (37.33)	1.85 (30.83)
Other healthcare services	HIV treatment services*	0–2
	No interruption		150 (50.00)	156 (52.00)	84 (28.00)	390 (43.3)
	Partial interruption		134 (44.67)	118 (39.33)	211 (70.33)	463 (51.44)
	Complete interruption		16 (5.33)	26 (8.67)	5 (1.67)	47 (5.22)
	Surgeries*	0–2
	No interruption		156 (51.83)	155 (51.67)	85 (28.33)	396 (44.00)
	Partial interruption		123 (41.00)	132 (44.00)	211 (70.33)	466 (51.78)
	Complete interruption		21 (7.00)	13 (4.33)	4 (1.33)	38 (4.22)
	Other services*§¶	0–2
	No interruption		102 (34.00)	172 (57.33)	115 (38.33)	389 (43.22)
	Partial interruption		149 (49.67)	117 (39.00)	57 (19.00)	323 (35.89)
	Complete interruption		49 (16.00)	11 (3.67)	128 (42.67)	188 (20.89)
	Total interruption score‡¶ (% with interruption)†	0–6	1.9 (31.60)	1.55 (25.83)	2.51 (41.83)	2.0 (33.33)
Total health service Interruption	Total interruption score‡ (% with interruption)†	0–20	6.2 (31.00)	5.4 (27.00)	7.9 (39.50)	6.49 (32.45)
	Low impact (total interruption score not above average)*		150 (50.00)	160 (53.33)	90 (30.00)	395 (43.90)
	High impact (total interruption score above average)*		150 (50.00)	140 (46.67)	210 (70.00)	505 (56.10)

* Number of observations (%).

† Mean score divided by the total score.

‡ Mean aggregated score.

§ Other services included outpatient clinics, referral, tuberculosis, and cancer care.

¶ Statistically different across countries at 95% significance level.

### COVID-19 and health service access from the perspective of the community.

A total of 1,797 adults from the community participated in this telephone survey; their average age was 42.3 years (Supplemental Table 1); males constituted 63% of the community participants. One-quarter (25%) of the community participants reported having difficulty accessing immunization services, and more than 20% reported having difficulty accessing preventive nutrition services or malnutrition management for their children during the COVID-19 pandemic. Access varied between sites, and the reported difficulty was greater at both sites in Nigeria. The mean interruption score for child healthcare was 0.87 out of 4. For maternal and reproductive care, 23% had difficulty accessing antenatal care, 22% had difficulty accessing iron and folic acid supplementation, and 20% had difficulty accessing family planning services. Similar to child services, the disruptions of maternal services were generally greater in Nigeria than in Burkina Faso and Ethiopia. For other health services, 18% had difficulty accessing HIV treatment and 14% had difficulty accessing surgeries (Table 3).

**Table 3:** Community residents’ responses to questions regarding the effects of COVID-19 on healthcare services in three sub-Saharan African countries

		Burkina Faso		Ethiopia		Nigeria		Total
		Nouna	Ouagadougou	Addis Ababa	Kersa	Ibadan	Lagos	
Having difficulty accessing child healthcare, count/total (%)	Immunization	86/278 (31)	17/202 (8.42)	34/220 (15.45)	46/256 (17.97)	57/147 (38.78)	75/155 (24.12)	315/1258 (25.04)
	Vitamin A	92/274 (33.58)	7/200 (3.50)	25/200 (12.50)	47/252 (18.65)	48/139 (34.53)	69/144 (47.92)	288/1209 (23.82)
	Nutrition preventive services	64/266 (24.06)	7/191 (3.66)	28/198 (14.14)	46/254 (18.11)	48/142 (33.80)	64/145 (44.14)	257/1196 (21.49)
	Malnutrition management	92/268 (34.33)	2/182 (1.10)	19/175 (10.86)	47/248 (18.98)	48/146 (32.88)	59/141 (41.84)	267/1160 (23.02)
	Access difficulty score‡ (out of 4), mean	1.16	0.16	0.46	0.72	1.39	1.66	0.87 (21.75%)¶
Having difficulty accessing maternal and reproductive care, count/total (%)	Antenatal care	95/270 (35.19)	5/182 (2.75)	20/182 (11)	40/232 (17.24)	40/130 (30.77)	54/133 (40.60)	254/1129 (22.50)
	Iron and folic	96/255 (37.65)	2/177 (1.1)	17/166 (10.24)	35/221 (15.84)	31/129 (24)	57/138 (41.3)	238/1086 (21.92)
	Family planning	84/247 (34)	4/204 (1.96)	18/185 (9.7)	39/224 (17.4)	27/131 (20.6)	52/140 (37)	224/1131 (19.81)
	Access difficulty score‡ (out of 3), mean	0.99	0.05	0.26	0.47	0.64	1.1	0.58 (19.33%)‡
Having difficulty accessing other healthcare, count/total (%)	HIV treatment	63/177 (35.59)	1/161 (0.62)	14/169 (8.28)	26/162 (16.05)	15/111 (13.51)	46/135 (34.1)	165/915 (18.00)
	Surgeries	40/187 (21.39)	1/186 (0.54)	18/191 (9.42)	19/147 (12.93)	18/122 (14.75)	38/146 (26.03)	134/979 (13.69)
	Other services*	0/268 (0)	7/242 (2.89)	7/263 (2.66)	7/166 (4.22)	3/158 (1.9)	5/183 (2.73)	29/1280 (2.27)
	Access difficulty score‡ (out of 3), mean	0.37	0.04	0.14	0.14	0.25	0.42	0.23 (7.67%)¶
Number of services with difficulty access (out of 10), mean†‡		2.45	0.21	0.73	1.3	1.5	2.2	1.4
Reduced access to care,§ count/total (%)¶		113/290 (38.96)	13/254 (5.12)	42/275 (15.27)	61/263 (23.2)	57/219 (26.03)	73/232 (31.46)	359/1533 (23.42)

* Other services included outpatient clinics, referral, tuberculosis, and cancer care.

† Aggregated mean score of reported access difficulty of all health services.

‡ Aggregated mean score.

§ Number with access difficulty above the average score.

¶ Mean score of access difficulty divided by the total access difficultly score.

### COVID-19 and prescription patterns.

**Figure 1.**
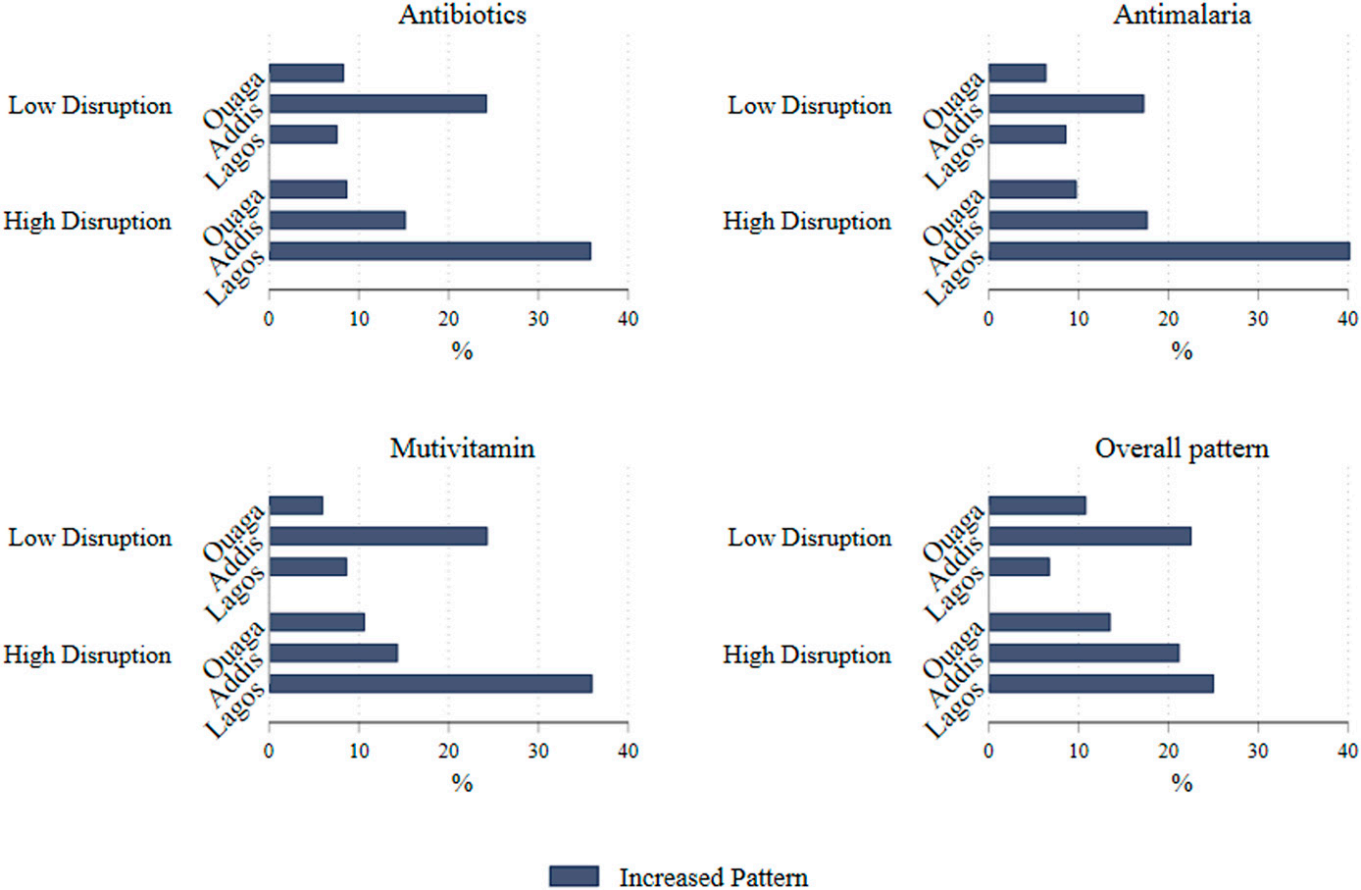
Percentage of healthcare providers who reported increases in prescription during the COVID-19 pandemic.

In Ethiopia, 36% and 31% of the HCPs reported increases in their prescriptions of antibiotics and antimalarial drugs, respectively. In Nigeria, 40% and 43% of the HCPs reported increases in their prescriptions of antibiotics and antimalarial drugs, respectively. In Ethiopia, 39% of the HCPs reported increases in their prescriptions of multivitamins. In Nigeria, 45% of the HCPs reported increases in prescriptions of multivitamins. Approximately 75% of the HCPs in Burkina Faso reported no change in prescribing antibiotics, antimalarial drugs, or multivitamins, whereas 54% of the HCPs in Burkina Faso reported no change in the prescriptions of other medications. The proportion of those reporting increased prescriptions was greater among HCPs whose services were highly disrupted than among those who reported low disruptions (Figure 1).

### Factors associated with service interruption caused by COVID-19.

Compared with the HCPs in Burkina Faso, the HCPs in Ethiopia had a 9% lower risk of experiencing high service interruptions (ARR, 0.91; 95% CI, 0.72–1.07), and the HCPs in Nigeria had a 38% higher risk of experiencing high service interruptions (ARR, 1.38; 95% CI, 1.19–1.59). Compared with physicians, nurses and other health providers had a 15% lower risk of reporting high service interruptions (ARR, 0.85; 95% CI, 0.56–0.95). Private health facilities and clinics and health outposts and centers had 29% (ARR, 0.71; 95% CI, 0.59–0.84) and 13% (ARR, 0.87; 95% CI, 0.66–1.16), respectively, lower risks of experiencing service interruptions compared with governmental institutions (Table 4).

**Table 4:** Factors associated with a high level of service interruption during the COVID-19 pandemic in three sub-Saharan African countries based on healthcare providers characteristics (*N* = 900)

	N (%)	CRR	95% CI	*P* value	ARR	95% CI	*P* value
Country							
Burkina Faso	150 (50)	Ref			Ref		
Ethiopia	134 (45)	0.89	0.75–1.09	0.19	0.91	0.72–1.07	0.28
Nigeria	221 (74)	1.4	1.29–1.68	< 0.001	1.38	1.19–1.59	< 0.001
Occupation							
Physicians	164 (59)	Ref	Ref
Nurses and other*	341 (55)	0.92	0.82–1.05	0.24	0.85	0.56–0.95	0.004
Facility							
Government	389 (62)	Ref			Ref		
Private	84 (41)	0.66	0.55–0.78	< 0.001	0.71	0.59–0.84	< 0.001
Others†	32 (47)	0.76	0.58–0.98	0.037	0.87	0.66–1.16	0.36
Treated COVID-19 patients							
Yes	301 (56)	1.01	0.90–1.14	0.87
No	204 (56)	Ref		
Workplace guidelines							
Yes	72 (41)	1.44	1.20–1.74	< 0.001	1.15	0.95–1.40	0.16
No	433 (60)	Ref			Ref		
Perceived stigma							
Yes	129 (54)	1.04	0.91–1.19	0.60
No	376 (57)	Ref	

Risk ratios were calculated using modified Poisson regression. ARR = adjusted risk ratio; CI = confidence interval; CRR = crude risk ratio.

* Clinical officers and community health workers.

† Public health and surgery.

## DISCUSSION

This study examined the impacts of the COVID-19 pandemic on health service disruptions in three sub-Saharan African countries from the perspectives of healthcare providers and community members. We observed substantial disruptions of essential health services during the COVID-19 pandemic. Child immunization and nutritional services and essential maternal and reproductive health services were particularly affected by the crisis. HCPs reported increased overall medication prescriptions, especially antibiotics and antimalarial drugs.

Sub-Saharan African countries have experienced constrained health systems and vulnerable economies even before the COVID-19 crisis; therefore, they were significantly affected by the pandemic.^29^ Our study showed that the COVID-19 pandemic had potentially detrimental impacts on the provision of essential health services in three countries across sub-Saharan Africa. More than half of the total essential health services, including child, maternal, and other services, were highly impacted by service interruptions caused by COVID-19. Such interruptions will likely have significant implications for long-term maternal, child, and adult health, and may more severely affect economically disadvantaged groups and lead to widened health inequities.

The impacts of the COVID-19 pandemic on healthcare services may vary from country to country, with the variations depending on the caseload of COVID-19 in each country and the robustness of a country’s healthcare systems before the pandemic. During our study, the interruptions of health services appeared exceptionally high in Nigeria compared with those in Burkina Faso and Ethiopia. This differential impact may be explained by the high number of active COVID-19 cases in Nigeria.^2,3^ For example, during August, Nigeria had 48,665, Ethiopia had 27,242, and Burkina Faso had 1237 total COVID-19 cases.^3,4^ These results suggest that a country’s pandemic curve affects health services impacted by the interruption of health services. When the burden of the COVID-19 caseload is greater, the impacts on access to other healthcare services will likely increase.

The interruptions of health services are also related to the specific services in question. During a global survey of the interruption of chronic medical health services, diabetes care was most affected by the reduction of healthcare resources caused by COVID-19, followed by chronic obstructive pulmonary disease, hypertension, heart disease, asthma, cancer, and depression.^30^ During this study, child healthcare appeared more interrupted than other services, including those related to maternal and reproductive health. This is in line with the results of a recent study performed in the United States that showed a decrease in the percentage of children who were up to date on their vaccinations during the COVID-19 pandemic.^31^ Other factors such as a lack of personal protective equipment, staff shortages, engagement of HCPs in COVID-19-related tasks, testing and surveillance, fear and stigma, and stay-at-home orders also could have disrupted the services. Different types of health facilities also appeared to experience differential impacts by the pandemic. During our study, compared with government hospitals and clinics, services provided by private hospitals were less affected by the COVID-19 pandemic. This finding may be explained by the fact that governmental hospitals tended to be the central hub for COVID-19 treatments and experienced greater pressure to shift their limited resources, the shutdown of community platform services events like village health and nutrition days to avoid crowding, and the shutdown of other essential services for the treatment of COVID-19.^29,32^

A substantial proportion of HCPs who participated in our study reported an increase in the prescriptions of medications during the COVID-19 pandemic, particularly antibiotics and antimalarial. This increase was observed more frequently for health services that were highly interrupted than for those that were not, suggesting that the more the health services are interrupted, the more likely it may be to secure antimicrobial medications without proper assessments and investigations. During the COVID-19 pandemic, even with the difficulty of interruptions of access to many health services, there appeared to be an increase in prescriptions of antibiotics and antimalarial drugs. The increase in antimalarial drug prescriptions could be attributable to the rainy season, which is when the data were collected. These antimalarial drugs also could have been prescribed for prophylactic purposes because they were highly promoted by politicians.^33^ This practice is alarming and its long-term ramifications for antimicrobial resistance require further investigation.^20^

To ensure the continued provision of critical child, maternal, and other health services during the COVID-19 crisis, innovative and adaptive measures are needed and the existing infrastructure available in a specific setting should be considered.^34^ Developed countries have rapidly opened COVID-19 testing and treatment centers and suspended nonessential health services and surgeries while maintaining the essential ones.^35^ Other measures used in developed countries include changing and adapting outpatient health services by using telemedicine, virtual healthcare, and digital technologies to continue routine services.^8^ Potential measures in sub-Saharan Africa may include strengthening the health system with systematic supply chain management of medicines and supplies, community engagement using mass media for the generation of health promotion, and remapping of the referral networks between facilities to maintain efficient patient flow to essential health services. The shifting and redistribution of tasks of healthcare providers and outreach services for chronic healthcare in the community may also prove useful for controlling the harmful effects of service interruption and reduced access. Community outreach programs that vaccinate children using a backlog with strict adherence to standard infection prevention will help control preventable morbidity and mortality of children. The adoption of telemedicine technologies that would assist the HCPs and facilities in providing high-quality health service is also recommended. Future studies should examine the long-term impacts of the COVID-19 pandemic on healthcare systems and the potential mitigation strategies to recover essential services in sub-Saharan Africa.

A key strength of this study was the inclusion of both HCPs and community members, allowing for the triangulation of findings from the perspectives of two groups of key stakeholders. The use of telephone-based surveys to enable remote and rapid data collection during the COVID-19 era was also a main strength of our study. We found that telephone-based surveys for healthcare and behavioral science studies were able to capture sensitive data points with high success rates. Limitations of the study included the opportunistic selection of the study sites, the potential nonresponses within sites, and the fact that some HCPs might work at the same facilities; all of these might have hampered the representativeness of the results to the broader national contexts. Health service interruption measurements through self-reports may potentially introduce some degrees of measurement error. In addition, the study did not include all health workers key to primary healthcare, such as community health workers, who are important for generating demand for essential services. However, we included multiple sites across sub-Saharan Africa and both rural and urban sites of the community members, which may have increased the generalizability of our findings to similar contexts.

In conclusion, this study contributes much-needed evidence of the impacts of the COVID-19 pandemic on the access and delivery of essential health services in sub-Saharan Africa. The findings of this study can be used by health authorities to create novel and adaptive strategies to recover and continue the provision of essential health services during the COVID-19 era.
